# Supplementary figures and images for: Visualization of endogenous G proteins on endosomes and other organelles
Source: eLife. 2024 Nov 8;13:RP97033. doi: 10.7554/eLife.97033 (PMC11548881; doi:10.7554/eLife.97033)

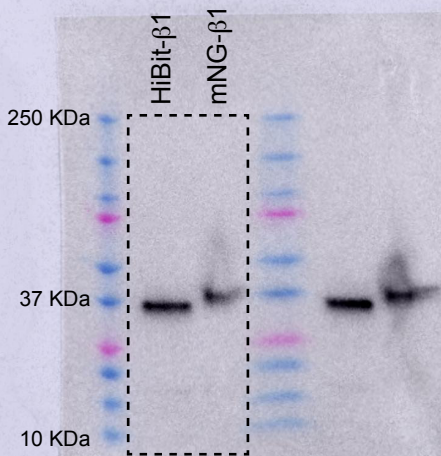

Supplement: Figure 1—source data 1. [file elife-97033-fig1-data1.pdf]

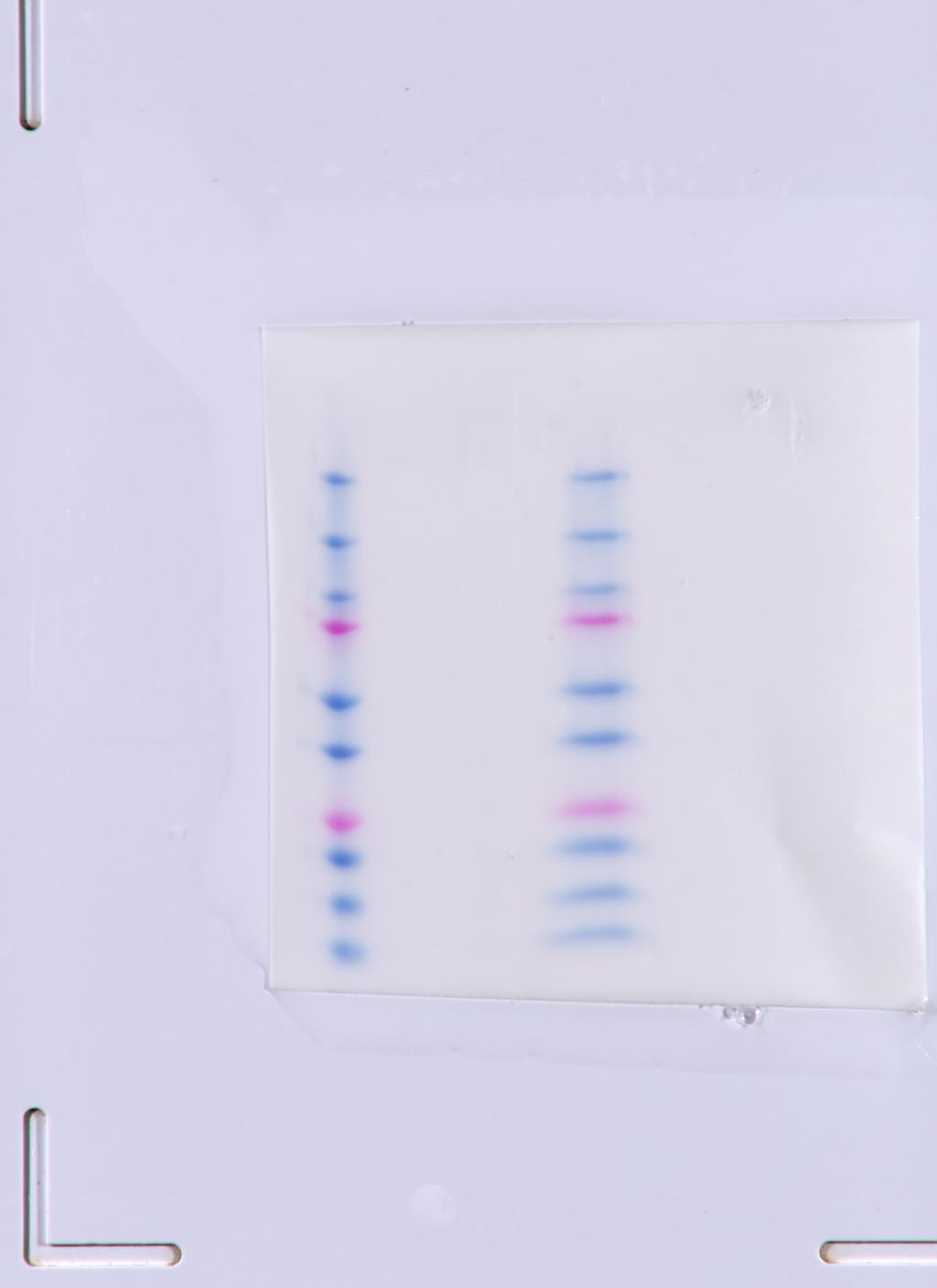

Supplement: Figure 1—source data 2. [file elife-97033-fig1-data2.zip › Figure 1ΓÇôsource data 2/09-12-23Gb1 36 33 1m 2023.09.12_08.39.06_Ch-Marker.jpg]

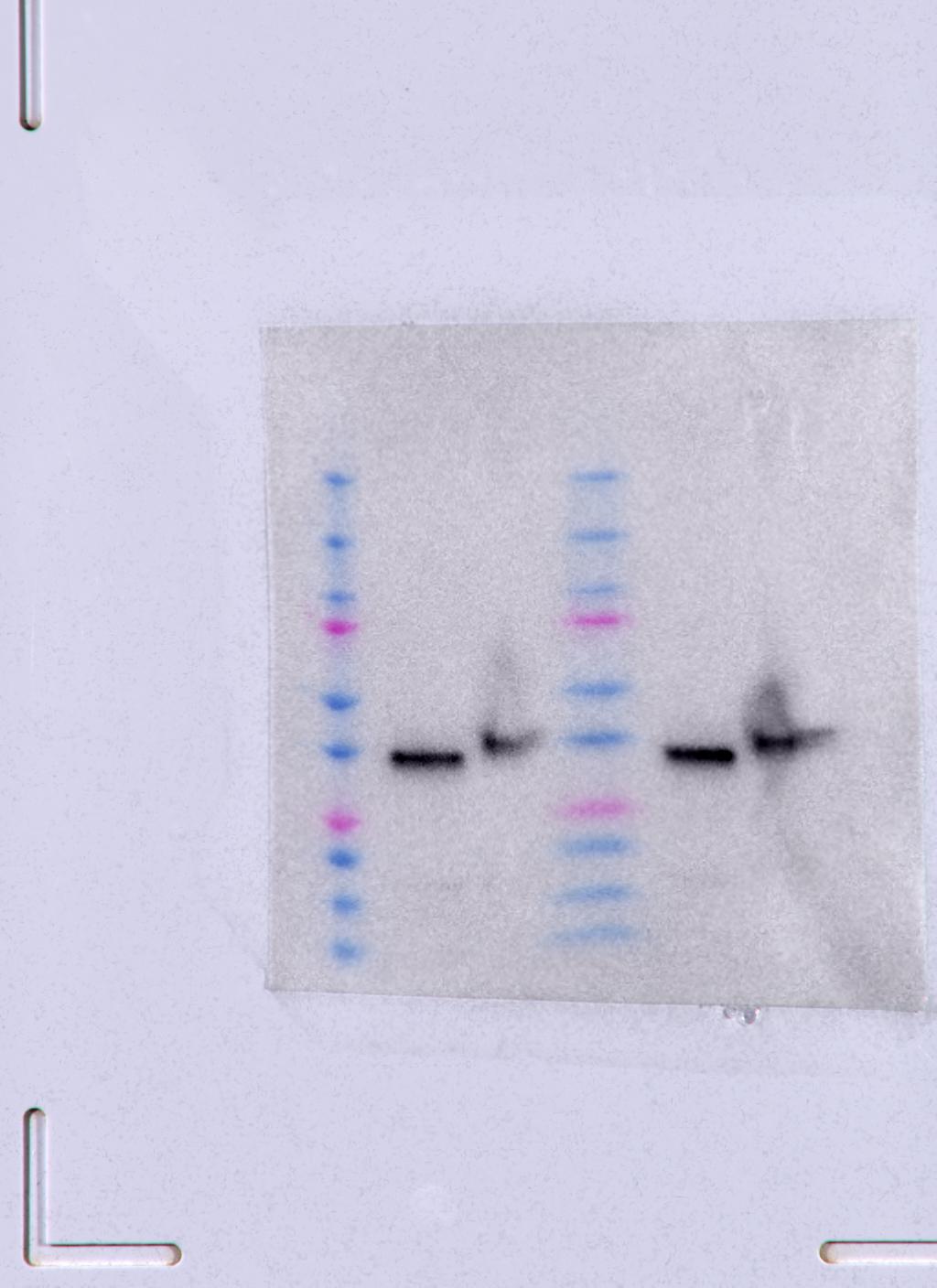

Supplement: Figure 1—source data 2. [file elife-97033-fig1-data2.zip › Figure 1ΓÇôsource data 2/09-12-23Gb1 36 33 1m 2023.09.12_08.39.06_Ch+Marker.jpg]

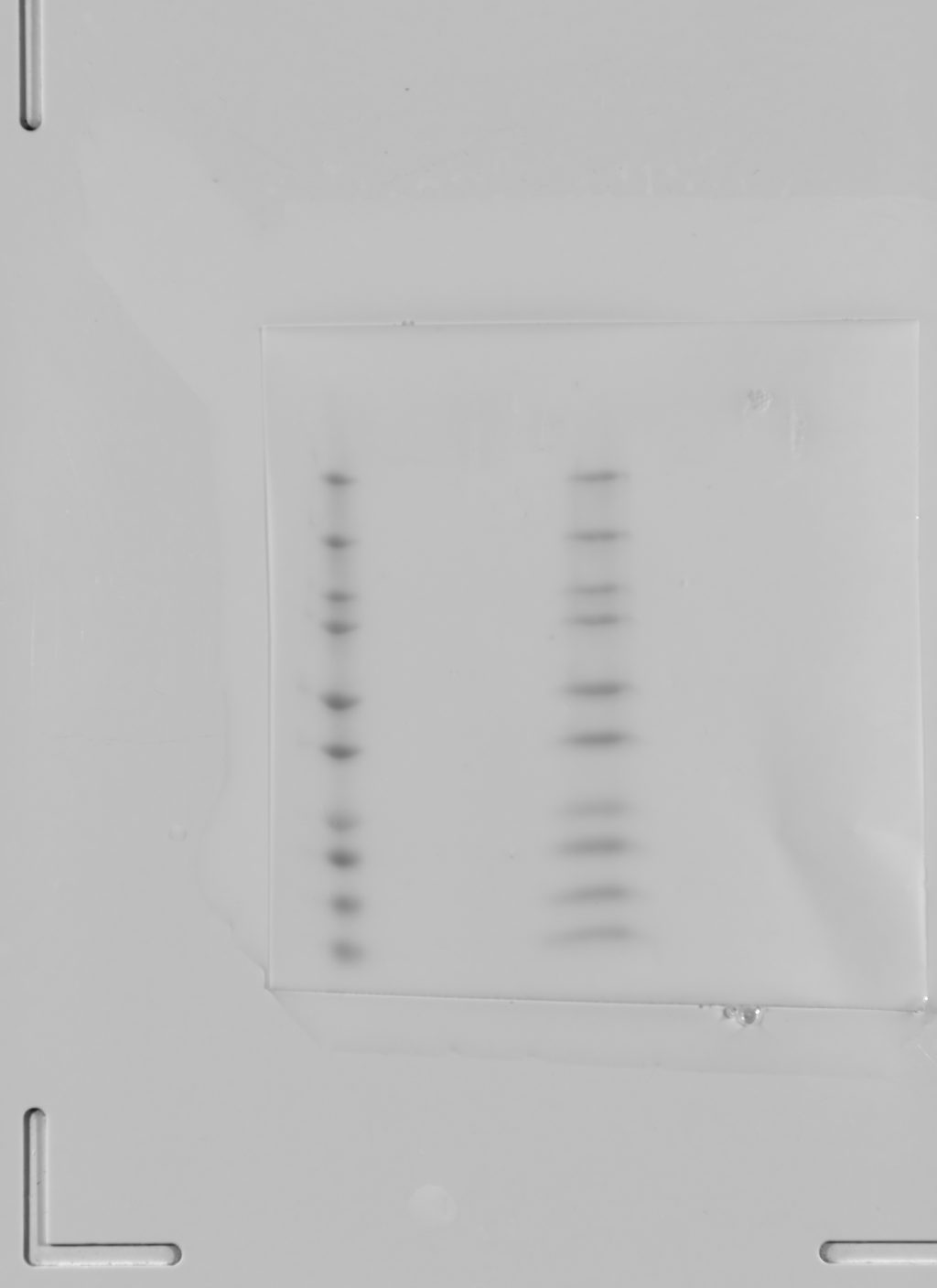

Supplement: Figure 1—source data 2. [file elife-97033-fig1-data2.zip › Figure 1ΓÇôsource data 2/09-12-23Gb1 36 33 1m 2023.09.12_08.39.06_Ch-Marker.tif]

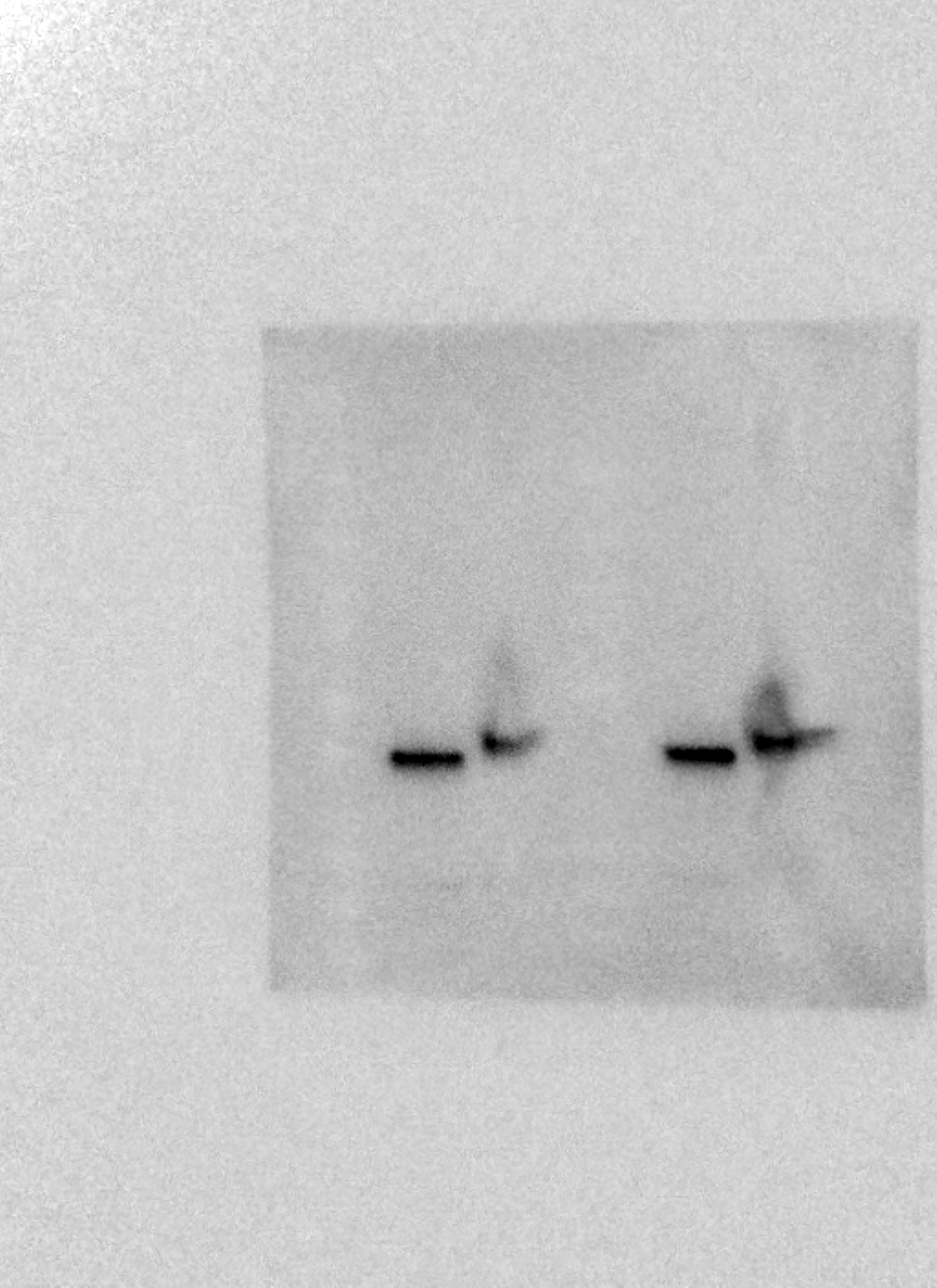

Supplement: Figure 1—source data 2. [file elife-97033-fig1-data2.zip › Figure 1ΓÇôsource data 2/09-12-23Gb1 36 33 1m 2023.09.12_08.39.06_Ch.tif]
